# Supplementary material for: An endogenous GLP-1 circuit engages VTA GABA neurons to regulate mesolimbic dopamine neurons and attenuate cocaine seeking
Source: Sci Adv. 2025 Feb 26;11(9):eadr5051. doi: 10.1126/sciadv.adr5051 (PMC11864183; doi:10.1126/sciadv.adr5051)
Supplement: Supplementary file 1 — Figs. S1 to S8 Legend for data file S1 [file sciadv.adr5051_sm.pdf]

Science Advances
AAAS

# Supplementary Materials for

## An endogenous GLP-1 circuit engages VTA GABA neurons to regulate mesolimbic dopamine neurons and attenuate cocaine seeking

Riley Merkel *et al.*

Corresponding author: Heath D. Schmidt, hschmidt@nursing.upenn.edu

*Sci. Adv.* **11**, eadr5051 (2025)
DOI: 10.1126/sciadv.adr5051

**The PDF file includes:**

Figs. S1 to S8
Legend for data file S1

**Other Supplementary Material for this manuscript includes the following:**

Data file S1

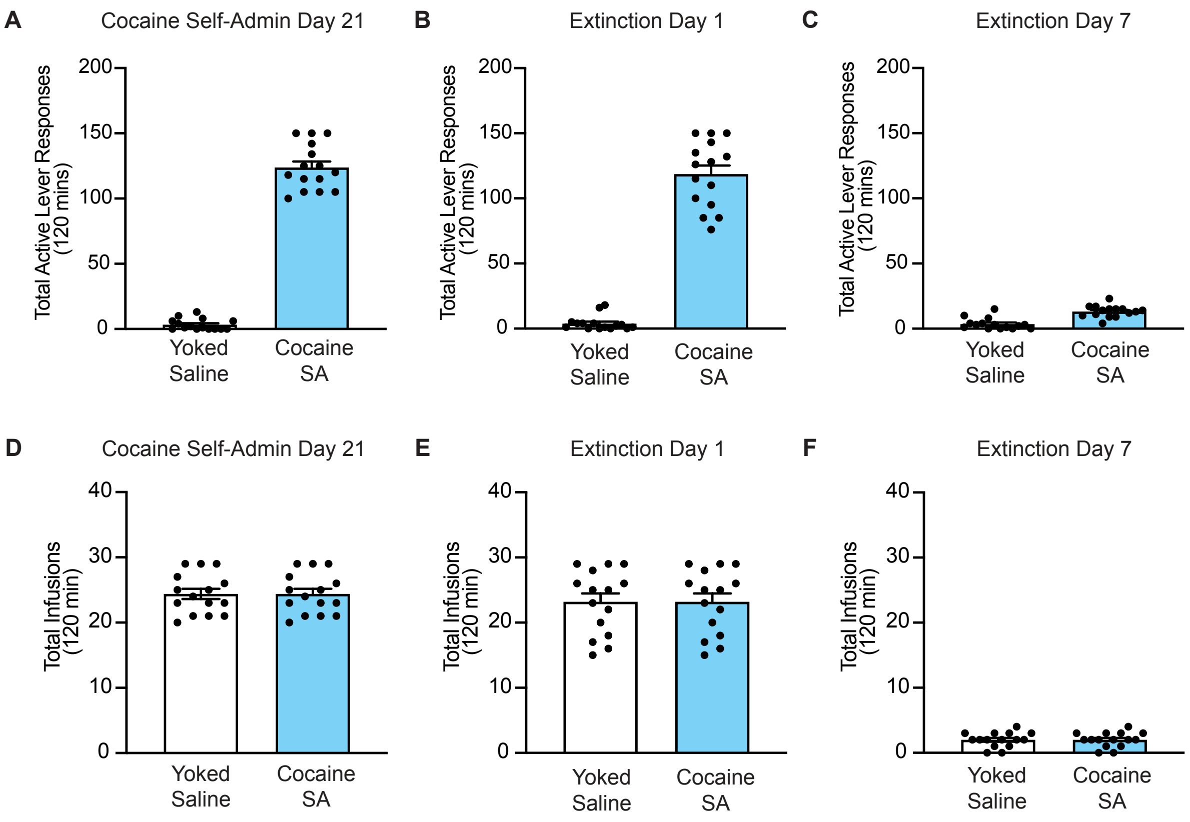


**Fig. S1. Behavioral data for cocaine self-administration and yoked-saline control rats at blood collection time points from Figure 1.** Total active lever responses for cocaine-experienced rats and yoked-saline control rats on self-administration day 21 (**A**), extinction day 1 (**B**), and extinction day 7 (**C**) (yoked saline: *n* = 15 (6 female & 9 male rats); cocaine self-administration: *n* = 15 (6 female & 9 male rats)). Total infusions for cocaine-experienced rats and yoked-saline control rats on self-administration day 21 (**D**), extinction day 1 (**E**), and extinction day 7 (**F**). Data are mean ± SEM.

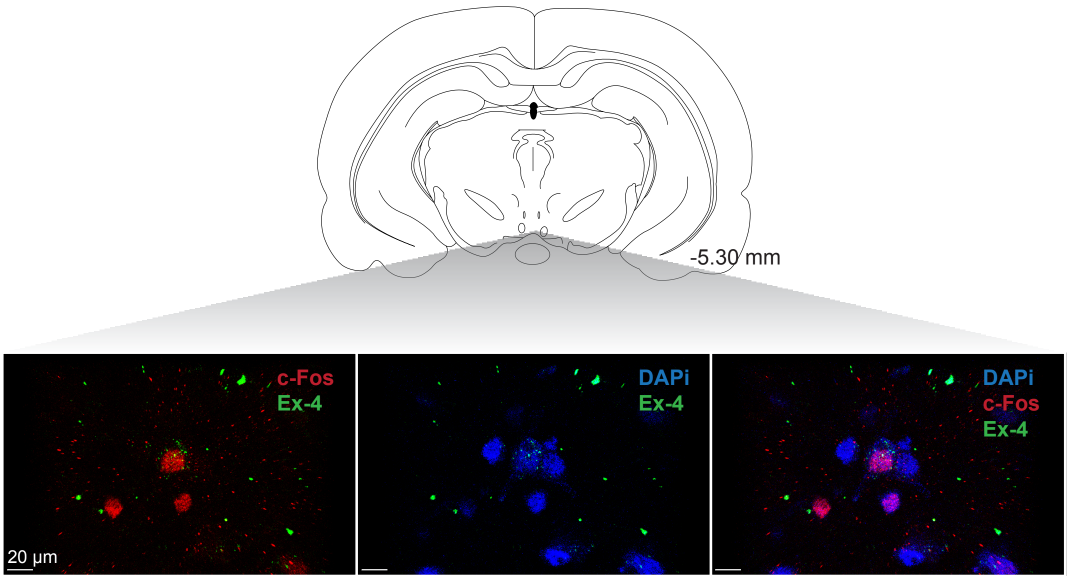


**Fig. S2. Systemically administered fluoro-exendin-4 binds to putative GLP-1Rs on neurons in the VTA and induces c-Fos expression.** A drug-naïve rat was pretreated with 0.2 µg/kg fluoro-exendin-4 (Ex-4; i.p.) and then sacrificed 90 minutes later. Immunohistochemistry identified fluoro-exendin-4 bound to VTA neurons expressing c-Fos [DAPi (blue), exendin-4 (green), and c-Fos (red)] (*n* = 1). Data are mean ± SEM.

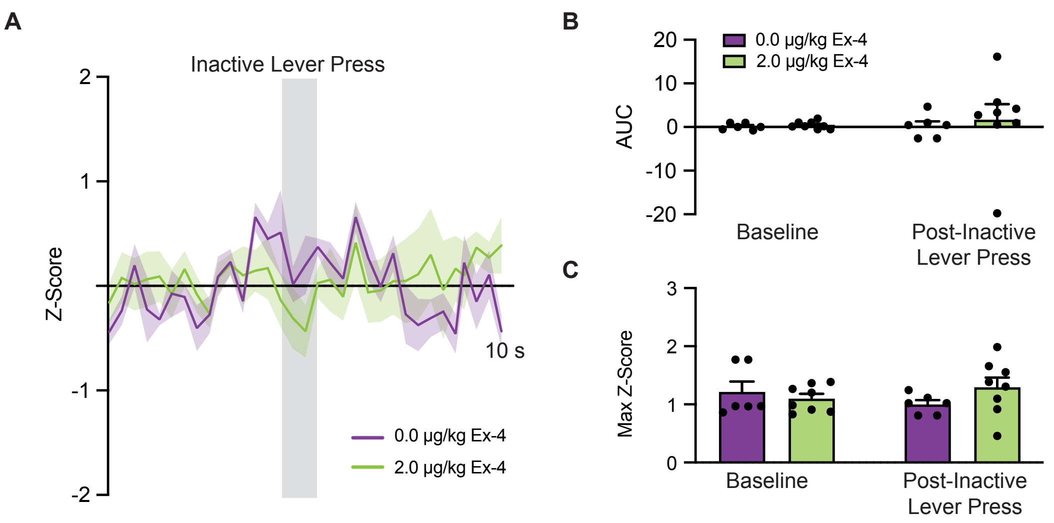


**Fig. S3. Systemic GLP-1R agonist pharmacotherapy does not alter VTA GABA neuron activity associated with inactive lever presses during cocaine reinstatement test sessions.** (**A**) Normalized z-score traces from inactive lever presses during reinstatement test sessions in cocaine-experienced rats pretreated with vehicle or exendin-4 (Ex-4) (*n* = 5 rats (3 female & 2 male rats); 2 inactive lever presses/rat/treatment). Some rats did not press the inactive lever during reinstatement tests (0.0 µg/kg exendin-4: *n* = 6 presses from 3 rats; 2.0 µg/kg exendin-4: *n* = 8 presses from 4 rats). (**B**) Responding on the inactive lever during reinstatement tests had no effect on the AUC of recorded $Ca^{2+}$ signals from VTA GABA neurons in cocaine-experienced rats treated with vehicle or exendin-4 (two-way ANOVA, treatment: $F_{1,24} = 0.1768$, $p = 0.6778$, time: $F_{1,24} = 0.1082$, $p = 0.7450$, treatment x time: $F_{1,24} = 0.07757$, $p = 0.7830$). (**C**) Responding on the inactive lever during reinstatement tests had no effect on maximum z-scores from VTA GABA neurons in cocaine-experienced rats treated with vehicle or exendin-4 (two-way ANOVA, treatment: $F_{1,24} = 0.4365$, $p = 0.5151$, time: $F_{1,24} = 0.005857$, $p = 0.9396$, treatment x time: $F_{1,24} =0.1452$, $p = 0.1452$). Data are mean ± SEM.

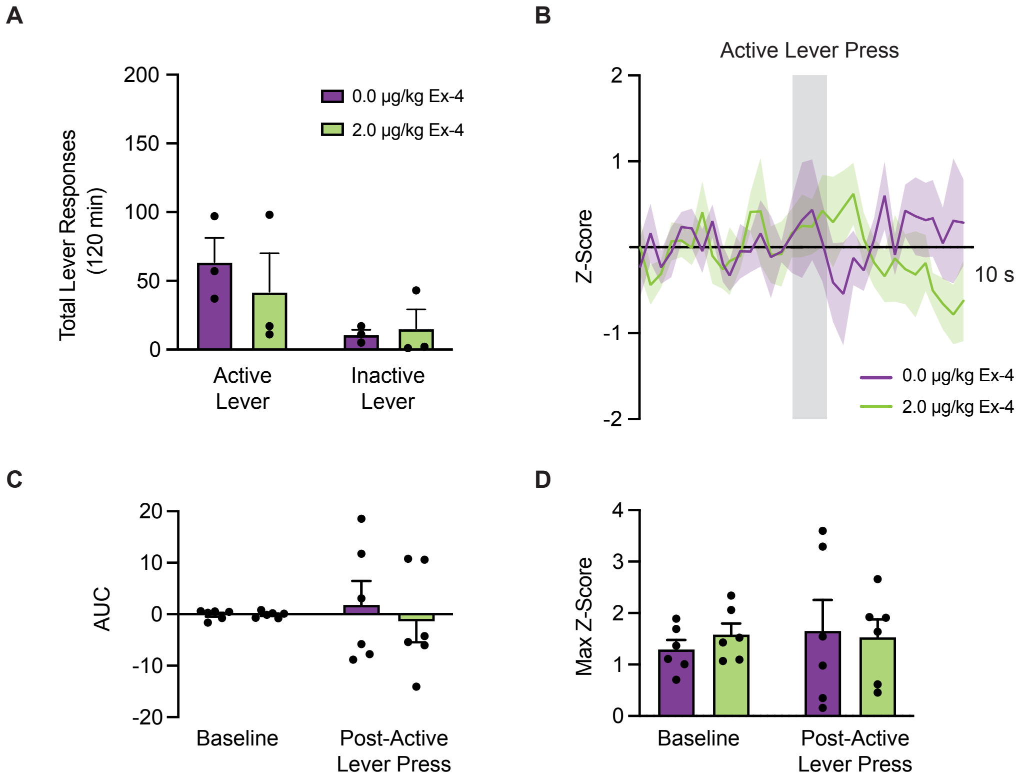


**Fig. S4. Systemic GLP-1R agonist administration does not alter VTA GABA neuron activity associated with active lever presses in drug-naïve rats.** **(A)** Systemic exendin-4 (Ex-4) administration had no effect on total lever responses during reinstatement test sessions in rats that previously self-administered saline (*n* = 3 (1 female & 2 male rats); two-way RM ANOVA, treatment: treatment: $F_{1,2} = 6.969$, $p = 0.1185$, lever: $F_{1,2} = 2.648$, $p = 0.2452$, treatment x time: $F_{1,2} = 0.7533$, $p = 0.4769$). (**B)** Normalized z-score traces during active lever presses in control rats pretreated with vehicle or exendin-4 (*n* = 3 rats; 2 active lever presses/rat/treatment). **(C)** Responding on the active lever had no effect on the AUC of recorded $Ca^{2+}$ signals from VTA GABA neurons in drug-naïve rats treated with vehicle or exendin-4 (*n* = 6; two-way RM ANOVA, treatment: $F_{1,10} = 0.2900$, $p = 0.6020$, time: $F_{1,10} = 0.008872$, $p = 0.9268$, treatment x time: $F_{1,10} = 0.2568$, $p = 0.6233$). (**D)** Responding on the active lever had no effect on maximum z-scores from VTA GABA neurons in drug-naïve rats treated with vehicle or exendin-4 (two-way RM ANOVA, treatment: $F_{1,10} = 0.06496$, $p = 0.8040$, time: $F_{1,10} = 0.1378$, $p = 0.7182$, treatment x time: $F_{1,10} = 0.2498$, $p = 0.6281$). Data are mean ± SEM.

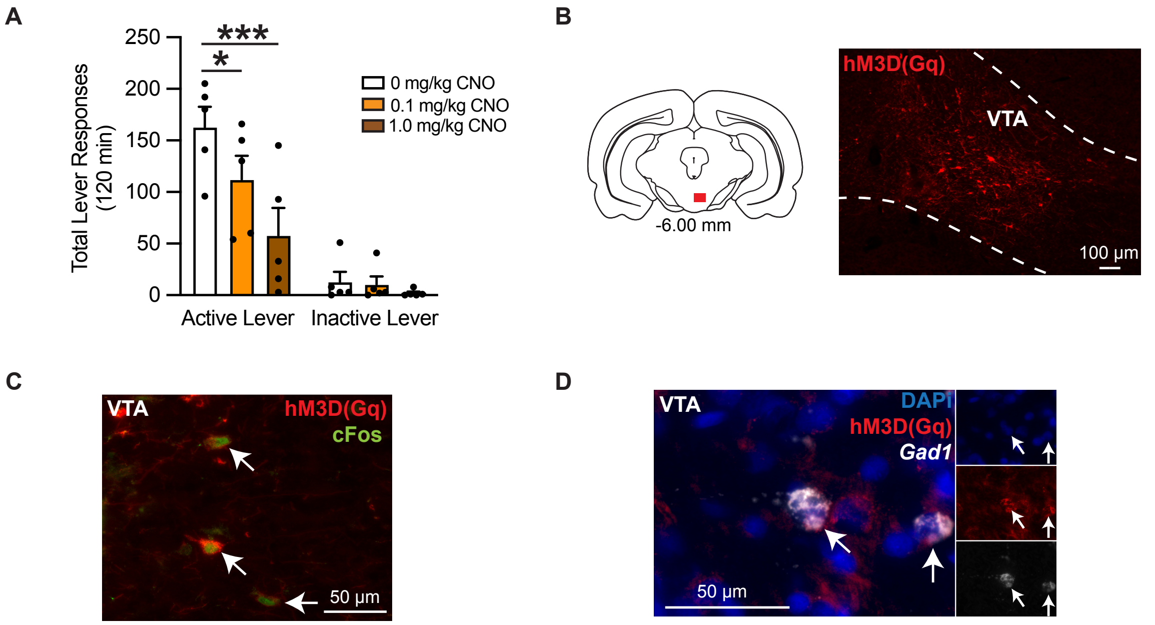


**Fig. S5. CNO dose-dependently attenuates the reinstatement of cocaine-seeking behavior in rats expressing hM3D(Gq) in VTA GABA neurons. (A)** Systemic CNO (0.1 and 1.0 mg/kg) significantly decreased active lever responses during cocaine reinstatement test sessions in rats expressing hM3D(Gq) in VTA GABA neurons ($n$ = 5; two-way RM ANOVA, treatment x lever: $F_{2,8}$ = 7.669, $p$ = 0.0138; Bonferroni's test: 0 mg/kg CNO vs 0.1 mg/kg CNO on active lever responses, *$p$ = 0.0342, 0 mg/kg CNO vs 1.0 mg/kg CNO on active lever responses, ***$p$ = 0.0005). (**B)** Representative image of hM3D(Gq) viral expression in the VTA. (**C)** Representative image showing c-Fos expression in hM3D(Gq)-expressing VTA neurons of rats treated with CNO. (**D)** FISH confirmed selective hM3D(Gq) expression in *Gad1*-positive cells [DAPi (blue), hM3D(Gq) (red), and *Gad1* (white)]. Data are mean ± SEM.

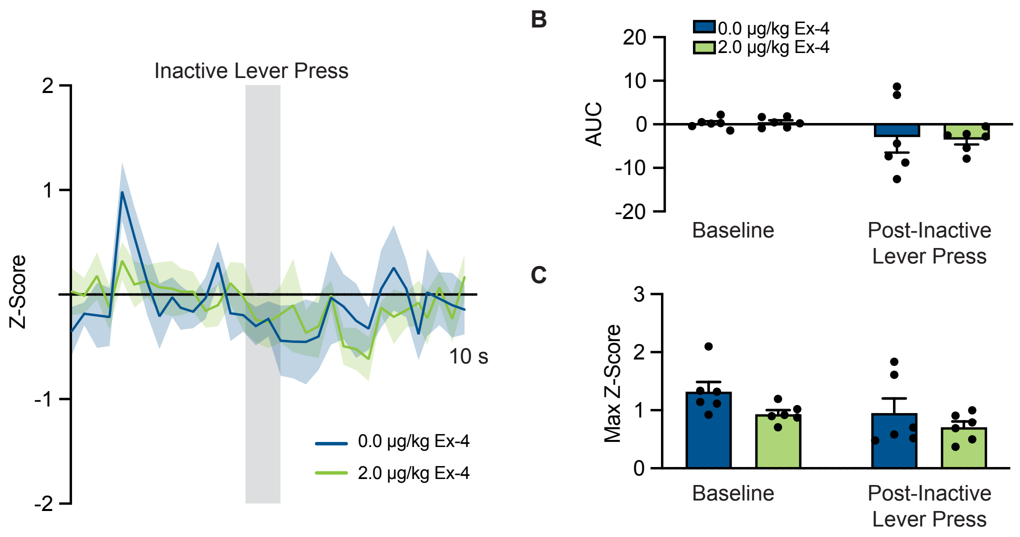


**Fig. S6. Systemic GLP-1R agonist pharmacotherapy does not alter VTA dopamine neuron activity associated with inactive lever presses during cocaine reinstatement test sessions. (A)** Normalized z-score traces from inactive lever presses during reinstatement test sessions in cocaine-experienced rats pretreated with vehicle or exendin-4 (Ex-4) (*n* = 5 rats (2 female & 3 male rats); 2 inactive lever presses/rat/treatment). Some rats did not press the inactive lever during recording (0.0 µg/kg exendin-4: *n* = 6 presses from 3 rats; 2.0 µg/kg exendin-4: *n* = 6 presses from 3 rats). **(B)** Responding on the inactive lever during reinstatement tests had no effect on the AUC of recorded $Ca^{2+}$ signals from VTA dopamine neurons in cocaine-experienced rats treated with vehicle or exendin-4 (two-way ANOVA, treatment: $F_{1,20} = 0.1161$, $p = 0.9153$, time: $F_{1,20} = 3.635$, $p = 0.0710$, treatment x time: $F_{1,20} = 0.04728$, $p = 0.8301$). (**C)** Responding on the inactive lever during reinstatement tests had no effect on maximum z-scores from VTA GABA neurons in cocaine-experienced rats treated with vehicle or exendin-4 (two-way ANOVA, treatment: $F_{1,20} = 3.891$, $p = 0.0625$, time: $F_{1,20} = 3.413$, $p = 0.0795$, treatment x time: $F_{1,20} = 0.1941$, $p = 0.6642$). Data are mean ± SEM.

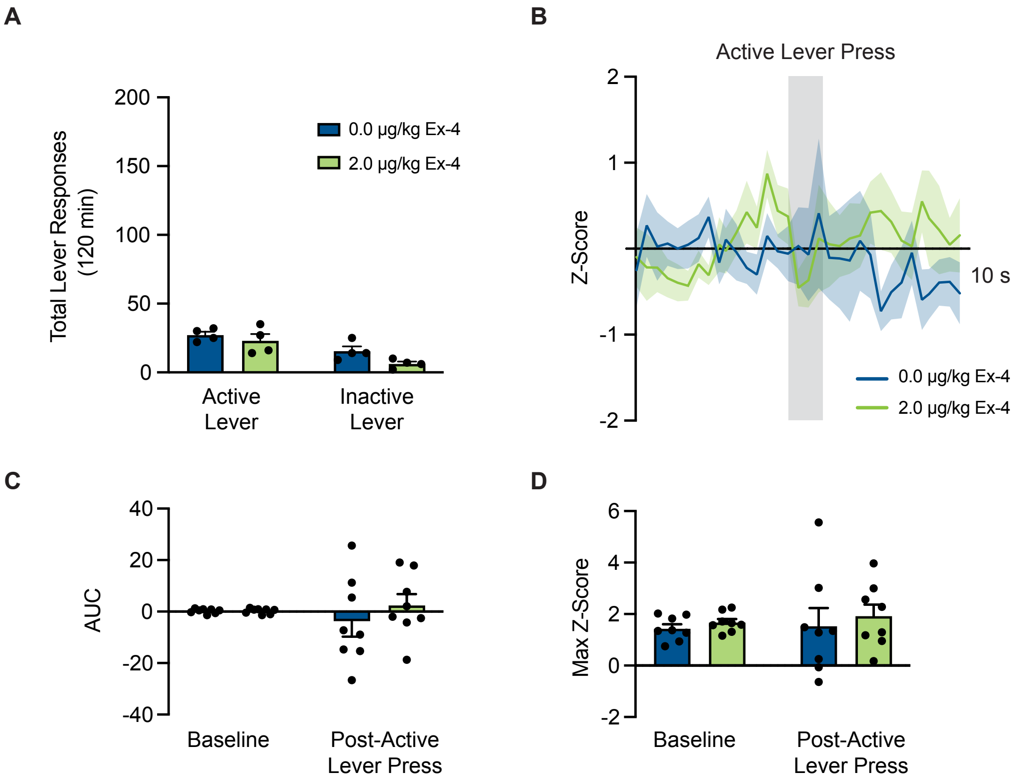


**Fig. S7. Systemic GLP-1R agonist administration does not alter VTA dopamine neuron activity associated with active lever presses in drug-naïve rats.** (**A**) Systemic exendin-4 (Ex-4) administration had no effect on total lever responses during reinstatement test sessions in rats that previously self-administered saline (*n* = 4 (2 female & 2 male rats); two-way RM ANOVA, treatment: treatment: $F_{1,3} = 5.622$, $p = 0.0984$, lever: $F_{1,3} = 9.976$, $p = 0.0509$, treatment x time: $F_{1,2} = 0.8876$, $p = 0.4156$). (**B**) Normalized z-score traces during active lever presses in control rats pretreated with vehicle or exendin-4 (*n* = 4 rats; 2 active lever presses/rat/treatment). (**C**) Responding on the active lever had no effect on the AUC of recorded $Ca^{2+}$ signals from VTA dopamine neurons in drug-naïve rats treated with vehicle or exendin-4 (*n* = 8; two-way RM ANOVA, treatment: $F_{1,14} = 0.7561$, $p = 0.3992$, time: $F_{1,14} = 0.05920$, $p = 0.8113$, treatment x time: $F_{1,14} = 0.6591$, $p = 0.4305$). (**D**) Responding on the active lever had no effect on maximum z-scores from VTA dopamine neurons in drug-naïve rats treated with vehicle or exendin-4 (two-way RM ANOVA, treatment: $F_{1,14} = 0.1463$, $p = 0.7078$, time: $F_{1,14} = 0.6045$, $p = 0.4498$, treatment x time: $F_{1,14} = 0.03093$, $p = 0.8629$). Data are mean ± SEM.

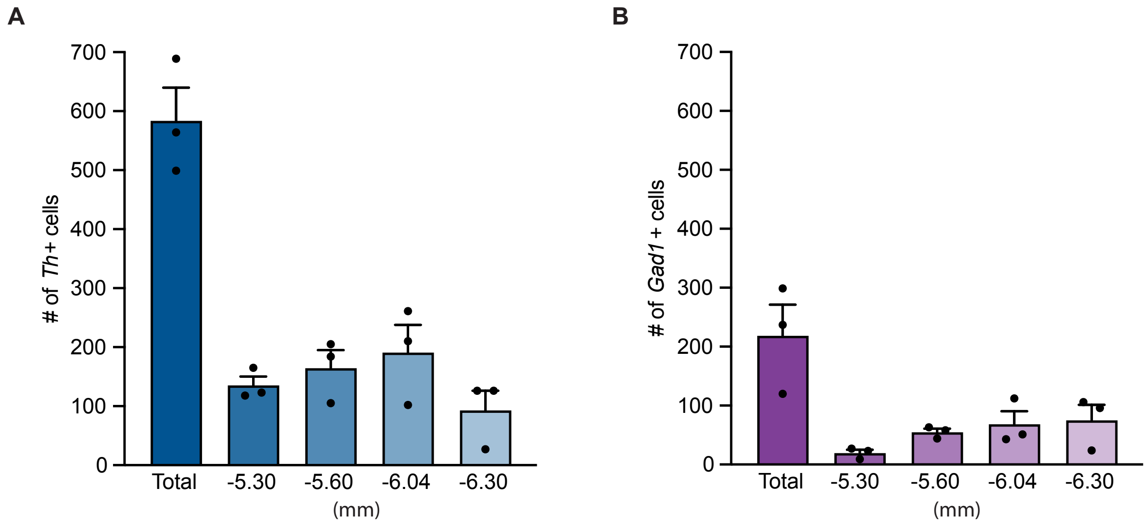


**Fig. S8. Distribution of *Th*+ and *Gad1*+ cells in the VTA.** The number of *Th*+ cells (**A**) and *Gad1*+ cells (**B**) in the VTA at different anterior/posterior positions relative to bregma ($n$ = 3 rats; 4 slices per rat). Data are mean ± SEM.

**Data “Figure 1A” to “Figure S8B”**

Separate file accessible at https://datadryad.org/stash/dataset/doi:10.5061/dryad.djh9w0w8v.

File contains all data points presented in the main and supplemental figures.
